# 非小细胞肺癌个体化治疗相关分子标记物

**DOI:** 10.3779/j.issn.1009-3419.2011.03.17

**Published:** 2011-03-20

**Authors:** 承泽 尉

**Affiliations:** 1 100048 北京，解放军总医院第一附属医院心胸外科 Department of Thraco-cardiac Surgery, the First Hospital Affiliated to General Hospital of the Chinese People's Liberation Army, Beijing 100048, China; 2 100850 北京，军事医学科学院307医院普通外科 Department of General Surgery, 307 Hospital of Academy of Military Medical Sciences, Beijing 100850, China

肺癌是人类发病率和死亡率最高的恶性肿瘤，全世界每年肺癌发生超过150万例，我国是肺癌的高发国家之一。2009年美国临床肿瘤学会（American Society of Clinical Oncology, ASCO）年会提出了“肿瘤个体化治疗，让癌症患者活得更长、活得更好”的主题，这也是近年来肿瘤治疗倡导的新理念，非小细胞肺癌（non-small cell lung cancer, NSCLC）患者的治疗更是需要这一原则，对每一个肺癌患者“量体裁衣”，根据肺癌组织的生物学特征及药物基因组学改变有针对性的进行治疗。本文对目前较为公认的与NSCLC肺癌个体化治疗相关的分子标记物进行综述，以期为NSCLC化疗有效药物的选择提供指导。

## 1 多耐药基因1（multidrug resistance gene 1, *mdr1*）

mdr1表达产物P糖蛋白（Pglycoprotein, P-gp）的过度表达是肿瘤细胞产生多药耐药的重要原因。mdr属于一个小的基因家族。在人类含有两个基因成员（*mdr1*, *mdr2*），而啮齿类动物则有3个基因成员（*mdr1*, *mdr2*, *mdr3*）。只有人类mdr1与啮齿类动物mdr1、mdr3可产生MDR现象。人类的mdr1定位于7q21.1，包括29个外显子和28个内含子，存在上游、下游两个启动子，编码长4.5 kb的mRNA，其翻译产物是P-gp。

P-gp是一个分子量为170 kDa的鳞酰化糖蛋白，有1, 280个氨基酸残基，整个分子由几乎完全相同的两个单体构成，每个单体具有6个跨膜区和1个ATP结合位点。跨膜区作为膜通道有利于物质转运；ATP结合位点位于胞内，为物质转运提供能量。

P-gp的功能包括：（1）P-gp是一种细胞膜ATP依赖泵，可结合并以耗能方式排出多种药物，降低细胞内药物浓度而产生耐药性。当抗癌药物进入耐药细胞后，P-gp即与抗癌药物结合，同时其ATP结合位点与ATP结合，水解ATP释放的能量主动地使药物从细胞内移至细胞外，导致细胞内抗癌药物浓度太低，不足以表现出细胞毒效应。P-gp的量随体外MDR细胞系耐药程度的增强而增加；（2）P-gp可能是钙离子通道的一部分。钙调蛋白抑制剂及其它钙通道阻滞剂可与P-gp结合，提高细胞内的药物浓度，增加MDR细胞对抗癌药物的敏感性；（3）mdr1编码生成的P-gp被认为是自然或人工环境中细胞防御毒物的机理^[1]^。P-gp主要位于细胞膜，具有的泵功能可向胞外排出食物中的天然毒物、内源性代谢产物和细胞毒性物质^[2]^。此外，P-gp也可表达于MDR细胞的核内和高尔基体，其功能与胞膜P-gp的功能相联系。

mdr1与肺癌预后不佳有关，mdr1因素造成的耐药细胞系在化疗期间扩增，导致药敏细胞减少，大多数转移的NSCLC对顺铂、卡铂、VP-16的化疗方案反应率低。随着对*MDR1*基因调控的研究的继续深入，有望为克服耐药寻找新希望。

## 2 肺耐药相关蛋白（lung resistance-related protein, LRP）

LRP是一种多耐药蛋白，首先发现于肺癌细胞中，故名为肺耐药蛋白。LRP蛋白是穹窿蛋白的主要成分，其过度表达明显影响药物的胞内转运和分布，导致靶点药物有效浓度下降而介导对铂类，烷化剂等耐药。目前认为其引起MDR的机制有2种：（1）它可以使那些以胞核为靶点的药物不能通过核孔进入细胞核，有些药物即使进入了细胞核内也会很快被运回到细胞质中，降低药物分布的核质比率；（2）可以使进入细胞质的细胞毒性或抑制性药物转运到运输囊泡中，呈房室性分布，使药物被隔离，最终通过胞吐的方式排出细胞外，从而产生肿瘤耐药。肺癌中LRP的高表达是预测化疗敏感性和预后的重要指标，阳性表达者对化疗敏感性差，化疗效果不好，预后差。

## 3 谷胱甘肽S转移酶（glutathion-S-transferase, GST-π）

GST-π首先由Mannervik从胎盘组织中提纯，GST-π在肿瘤病变中异常表达作为一种肿瘤标志物，目前已引起许多学者的重视。GST-π是在体内生物转化过程中催化致癌物或药物等亲电子剂与还原型谷胱甘肽结合反应的生物转化酶，具有代谢、转运药物及解毒等功能，可保护正常细胞免受细胞毒性物质的损害。GST根据等电点可分为GST-α、GST-μ、GST-π三种，GST-π不易被化学物质诱导，但在化学致癌物质诱发癌变和增生结节中则明显增加。GST-π对包括致癌剂在内的细胞毒性因子具有一定的抵抗力，从而保护DNA免受损伤。正常肺组织GST-π为阴性，而肺癌及癌旁肺组织GST-π大量表达，说明此酶正处于激活状态，从而达到清除致癌物质，保护DNA遗传物质稳定的目的。在肺癌治疗中，肿瘤是否对化疗药物敏感，GST-π起到一定作用，通过抑制肿瘤细胞内GST-π的活性，可提高癌细胞对药物化疗效果。

GST阳性表达和顺铂、阿霉素耐药相关，但与丝裂霉素和长春新碱耐药的关系不明显。GST的过表达通常与MDR1表达增加相关。对耐顺铂的肺癌细胞的研究中发现，细胞质内的谷胱甘肽和过氧化氢酶含量增高，但抑制过氧化氢酶后药物敏感性无明显提高，而抑制谷胱甘肽后药物敏感性明显提高。对化疗前后肺癌细胞耐药的研究发现，与化疗前细胞株相比，化疗后细胞株对阿霉素的耐药性增加3倍，对顺铂的耐药性增加217倍，但未见*MDR1*基因表达，而GST水平明显升高，说明其化疗耐药性与GST活性有关^[3]^。

## 4 胸苷酸合成酶（thymidlylate synthase, TS）

人类*TS*基因定位于第18号染色体上（18pl1.3）。是由两个相同亚单位组成的二聚体蛋白，每个亚单位的分子量为36 kDa。*TS*基因编码的胸苷酸合成酶是DNA合成的关键酶，在DNA合成与修复中起重要作用，也是叶酸代谢循环中起中心作用的酶类之一。氟脲是胸苷酸合成的主要抑制剂之一，广泛用于肿瘤的化疗，其代谢产物与TS形成络合物，干扰DNA合成从而杀死肿瘤。TS是5-Fu为基础化疗的靶向酶。*TS*基因多态性导致其在肿瘤细胞中的表达效率不同。因此研究*TS*基因型与蛋白表达、肿瘤发生、发展及化疗药物敏感性的关系对肿瘤的预防和治疗具有重要指导意义。TS高表达可见于高分化肿瘤。同时，临床前研究^[4]^显示TS表达增高与培美曲塞的敏感性降低相关。因此，在肿瘤组织中，*TS*基因表达增高预示患者对培美曲塞耐药。

## 5 核糖核苷酸还原酶M1（ribenucleotide reductase M1, RRM-1）

*RRM-1*是编码核糖核苷酸还原酶调节亚基的基因，在核苷酸转变为脱氧核苷酸的过程中起着至关重要的作用。核糖核苷酸还原酶（ribenucleotide reductase M, RRM）是DNA合成途径的限速酶，在DNA合成修复途径中发挥重要作用。它主要催化二磷酸核糖核苷酸转化为二磷酸脱氧核糖核苷酸，由M1、M2两种亚型构成，M1亚型控制其底物特异性及整体酶活性的开关；M2主要携带与底物转换有关的催化区域。在完全切除的、未接受围手术期化疗或放疗的NSCLC中，RRM-1 mRNA水平是生存预后指标，肿瘤RRM-1 mRNA高表达的患者生存明显长于低表达者^[5]^。国外研究^[6]^表明，RRM1表达与吉西他滨耐药密切相关。RRM1不仅是吉西他滨疗效的敏感分子，也是肿瘤的独立预后影响因子。有研究^[7]^发现187例早期NSCLC中，与RRM1低表达者（60.2个月）相比，高表达者总生存期（> 120个月）明显延长。

## 6 β-微管蛋白（tubulin-β）

微管蛋白分为α、β2个亚型，为细胞骨架的重要组成部分，它担任着维持细胞形态、进行物质交换、传递信息以及参与有丝分裂等重要功能。紫杉醇可以促进微管蛋白装配成微管，但抑制微管的解聚，从而导致微管束的排列异常，形成星状体，使纺锤体失去正常功能，导致细胞死亡。最近的一项多中心随机研究^[8]^显示采用吉西他滨/cDDP、长春瑞滨/cDDP和紫杉醇/卡铂治疗NSCLC患者在疾病进展时间（time to progression, TTP）方面无差别。这些药物主要的耐药机制是特定微管蛋白的基因多态性或同型过度表达，特别是氨基酸残基1-31和217-233，它们在紫杉醇与微管蛋白的结合过程中发挥着重要作用，而在结合位点附近的突变，如β-微管蛋白的Thr274Ile和Arg282Gln，可能与耐药产生有关^[9]^。因此，微管蛋白序列变异分析和同型抗原表达测定对评估紫杉烷类疗效非常重要。β-微管蛋白CLASS1突变已经在NSCLC患者中被发现：49例NSCLC患者中有33%被检测到该突变，对紫杉醇治疗均无反应^[10]^。β-Ⅲ微管蛋白同种型表达上调是耐药产生的另一重要原因，β-Ⅲ微管蛋白浓度增高与紫杉醇类药物的敏感性降低相关^[11]^。在紫杉醇/卡铂组的患者中β-Ⅲ微管蛋白低水平可以获得更好的疗效，并且β-Ⅲ微管蛋白表达水平和TTP也有相互关联的趋势。

长春瑞滨和紫杉醇作用相反，它能够促进微管解聚，抑制细胞内微管蛋白的聚合，阻止增殖细胞有丝分裂过程中纺锤体的形成，使细胞分裂停于有丝分裂中期。在联合用药过程中，与药物代谢密切相关的代谢酶的突变可能会改变长春瑞滨和其它化疗药物的药物活性。Sere等^[12]^研究了93例Ⅲ期、Ⅳ期NSCLC的β-Ⅲ微管蛋白表达与以长春瑞滨为基础化疗德疗效的关系，结果发现，β-Ⅲ微管蛋白表达水平与化疗有效率无关，但高表达患者的总生存期和进展前生存期明显降低，是其独立的预后因子。同样的结论在Dumontet^[13]^的研究中也得到证实。这表明β-Ⅲ微管蛋白的表达程度影响NSCLC对紫杉烷类及长春瑞滨的敏感性，可以指导个体化疗方案的制定。

## 7 切除修复交叉互补基因1（excision repair cross complement-1, *ERCC-1*）

顺铂耐药包括内源性或获得性，包含两种机制：（1）减少细胞毒的铂-DNA复合物形成；（2）铂-DNA复合物形成防止细胞死亡，铂类药物是DNA交联药物的一种，其抗肿瘤作用可能与铂-DNA链间交联关系不大，而与铂-DNA链内交联关系更密切，链内交联主要依赖核酸切除修复（nucleotide excision repair, NER）系统修复^[14]^。NER系统是存在于大肠杆菌，哺乳动物中负责修复DNA链内损伤的系统，是包含*XPO-HHRAD23*、*XPA*、*BPA*、*THIIH*、*XPG*、*XPB*、*XPD*、*ERCC1-XPF*、*RFC*、DNA连接酶等在内的一大组基因，其中*ERCC-1*作为损伤碱基5’-3’的切割酶起重要作用。目前NER被描述成五步模式：损伤的DNA由多蛋白复合物识别；在损伤部位的两端引入切割酶；切下损伤的核苷酸片段；以互补链为模板，合成一小片段核苷酸；封闭两端的切口完成修复全过程。在上述DNA损伤的切除修复过场中*ERCC-1*是NER途径的关键基因，*RECC-1*具有损伤DNA 5’识别的功能，而且具有5’-3’核酸内切酶的活性。若缺乏*ERCC1*，泡状链内铂-DNA加合物的修复大大受限，从而使化疗敏感性明显增加；相反若*ERCC1*表达增加，则DNA修复能力增加，从而使化疗敏感性下降，表现为铂类耐药。

Ryu等^[15]^研究发现ERCC1第118位密码子的多态性可影响患者的生存率和疗效，基因型为C/T或T/T的患者中位生存时间（median survival time, MST）为281 d，而基因型为C/C的患者的MST为486 d，提示*ERCC1*第118位密码子可作为预测以顺铂为基础治疗NSCLC患者疗效的指标。Ceppi等^[16]^通过对接受顺铂治疗的NSCLC患者的检测发现ERCC1 mRNA低表达患者的MST为23.0个月，而高表达者为12.4个月（*P*=0.000, 1）。Isla等^[15]^分析了106例晚期NSCLC中*ERCC1*基因的单核苷酸多态性，发现ERCC1C8092A基因型明显影响泰索帝卡铂的疗效，以C/C基因型患者MST最大。Soria等^[17]^通过免疫组化的方法检测了术后辅助化疗临床试验IALT研究（International Adjuvant Lung Cancer Trail）的761例肺癌手术标本发现，335例（44%）患者的*ERCC1*阳性，含顺铂方案辅助化疗受益情况与ERCC1状态有关（*P* < 0.009），在*ERCC1*阴性的患者中辅助化疗组的MST较单纯手术组增加了14个月（HR=0.67, *P* < 0.006）；*ERCC1*阳性的患者则不能从辅助化疗中临床获益（HR=1.18, *P*=0.29），首次在大样本研究中证实了功能基因对肺癌个体化治疗的指导作用。目前认为NSCLC根治术后肿瘤组织中*ERCC1*检测阴性的患者应该接受辅助化疗，并可从中获得生存收益。

## 8 表皮生长因子受体（epider growth factor receptor,*EGFR*）

*EGFR*基因是ErbB家族成员之一，位于染色7p12-14区。*EGFR*基因酪氨酸激酶功能区由外显子18-24编码，在肺癌中90%以上的*EGFR*基因突变集中在外显子18-21，其中特征性突变主要有：（1）外显子18点突变，719密码子甘氨酸错义突变为半胱氨酸（G719S）；（2）外显子19缺失，主要是编码氨基酸（KELREAT）序列缺失，这一缺失改变了EGFR受体ATP结合巢（ATP-binding cleft）的角度，增强了肿瘤细胞对小分子酪氨酸激酶抑制剂的敏感性^[18]^；（3）外显子20的点突变或碱基插入突变，点突变主要是第790位密码子出现C-T转换，引起EGFR蛋白中该位点的氨基酸由苏氨酸转变为甲硫氨酸（T790M），这一突变见于EGFR酪氨酸激酶抑制剂（EGFR tyrosine kinase inhibitors, EGFR-TKIs）治疗后复发者，突变使得肿瘤细胞对吉非替尼或厄洛替尼产生抗性；碱基插入突变出现在第770-775位密码子，存在8种不同的插入方式，插入的片段为3个-9个碱基。这类插入突变的临床意义目前尚不清楚^[19-21]^；（4）外显子21点突变，858位密码子亮氨酸置换成精氨酸（L858R），位于DGF序列附近，其作用使A-loop的稳定性增强，提高了肿瘤细胞对EGFR-TKIs的敏感性。

*EGFR*基因突变可明显提高EGFR-TKIs的临床反应率。临床研究^[22]^表明：*EGFR*突变患者接受吉非替尼或厄洛替尼治疗的有效率较高。*meta*分析^[23]^证实*EGFR*突变为吉非替尼疗效的分子预测标志。因此*EGFR*突变是预测EGFR-TKIs治疗有效率强有力的预测因素，肺癌患者接受靶向治疗之前应进行*EGFR*基因突变检测。

不同的*EGFR*突变类型可预示EGFR-TKIs临床疗效的差异。Jackman等^[24]^对*EGFR*基因突变NSCLC患者进行的亚组分析结果表明，*EGFR*外显子19缺失患者总生存期及到进展/死亡时间都长于*EGFR*外显子21 L858R点突变者，且前者在症状改善方面也优于后者。

## 9 *KRAS*基因突变

*KRAS*基因是*EGFR*下游的关键调节分子，NSCLC中*KRAS*基因突变约占20%。Raponi等^[25]^报道NSCLC中*KRAS*基因突变对抗-EGFR治疗的阴性预测值为97%，因此*KRAS*基因突变可用于排除NSCLC患者的抗-EGFR治疗，在EGFR-TKIs治疗前，*KRAS*基因突变作为常规检测指标；*KRAS*基因突变常提示患者预后不良，并对吉非替尼和厄洛替尼的治疗有抗性，*KRAS*基因突变与肺癌患者对TKIs的原发耐药有关^[26-29]^。*KRAS*基因突变是*EGFR*靶向治疗的负性预测因子。

## 10 p53

p53蛋白对细胞周期的调控，细胞凋亡、存活、基因转录和DNA修复均非常重要。Tsao等^[30]^对JBR.10试验的患者术后标本进行了检测，入选的患者均为Ⅰb和Ⅱ期的NSCLC，术后接受4个周期的长春瑞滨联合顺铂的治疗观察，在可获得标本的253例患者中，132例（52%）p53蛋白高表达，未接受化疗的p53(+)的患者总体生存明显短于接受治疗的p53(-)的患者（*P*=0.03），p53(+)的患者更易从辅助化疗中得到生存收益（HR=0.54, *P*=0.02），而*p53*基因有无突变则与预后及治疗是否获益无关，研究者认为p53蛋白过表达不仅提示预后不佳，而且提示完全切除的NSCLC患者能从辅助化疗中获益。

## 11 血管内皮生长因子（vascular endothelial growth factor, VEGF）及其受体（VEGF reportor, VEGFR）

血管生成在肺癌的形成、生长和转移过程中发挥着重要作用，血管生成受到多种细胞因子和生长因子的调控，其中VEGF及VEGFR在促进肺癌新生血管生成过程中起关键作用。VEGF家族包括VEGF-A、VEGF-B、VEGF-C、VEGF-D。VEGF-A主要调节血管的生成；VEGF-B在结直肠癌的转移淋巴结中有高水平表达；VEGF-C和VEGF-D主要调节淋巴管的生成。VEGF生物活性依靠其与特异受体的反应，VEGFR家族包括VEGFR1、VEGFR 2和VEGFR 3。血管生成抑制剂通过抑制肺癌血管生成，进而诱导肺癌细胞凋亡，抑制肺癌生长和复发。目前以血管生成相关基因为靶点的药物有贝伐单抗和重组人血管内皮抑制素注射液。

贝伐单抗是针对VEGF的重组人源化单克隆抗体，可以封闭VEGF，使VEGF不与其受体相结合，从而阻断VEGF导致的血管通透性的增加，并抑制肿瘤的生长。最近的AVAiL研究^[31]^进一步证实了ECOG-4599研究^[32]^提出的观点：贝伐单抗联合化疗能够明显提高非鳞癌的Ⅲb期/Ⅳ期NSCLC患者的无疾病进展生存期和客观有效率，给患者带来临床获益。

目前由于NSCLC的发生和发展过程尚未完全明确，根据分子标志来指导NSCLC的治疗目前尚于初级阶段，将功能基因组学、功能蛋白组学和其分子生物学结合起来，对不同基因和各传导通路的进一步深入认识，从而有望于将理论转向临床实际运用。在研究中发现了一些分子标志物，具有预测或预后的作用。有预测作用的分子标记物与治疗方案相关，尚需大规模的前瞻性研究来进一步的区分和佐证，以期实现患者的个体化治疗。
